# Bioactive Compounds in Potato Tubers: Effects of Farming System, Cooking Method, and Flesh Color

**DOI:** 10.1371/journal.pone.0153980

**Published:** 2016-05-03

**Authors:** Magdalena Grudzińska, Zbigniew Czerko, Krystyna Zarzyńska, Monika Borowska-Komenda

**Affiliations:** 1 Division of Jadwisin, Plant Breeding and Acclimatization Institute–National Research Institute, Jadwisin, Poland; 2 Section of Agricultural Chemistry, Department of Soil Environment Sciences, Faculty of Agriculture and Biology, Warsaw University of Life Sciences-SGGW, Warsaw, Poland; Agriculture and Agri-Food Canada, CANADA

## Abstract

We investigated the effect of cultivation system (conventional or organic), cooking method, and flesh color on the contents of ascorbic acid (AA) and total phenolics (TPs), and on total antioxidant activity (Trolox equivalents, TE) in *Solanum tuberosum* (potato) tubers. The research material, consisting of 4 potato cultivars, was grown in experimental fields, using organic and conventional systems, at the experimental station in 2012 and 2013. The analysis showed that organically grown potatoes with creamy, light yellow, and yellow flesh had significantly higher TPs than did potatoes grown conventionally. Flesh color and cooking method also affected AA. The greatest losses of AA occurred in yellow-fleshed potatoes grown conventionally and cooked in the microwave; such losses were not observed in potatoes grown organically. A dry cooking method (baking in a microwave) increased the TP contents in potatoes by about 30%, regardless of the flesh color and the production system. TE was significantly higher in organically grown potatoes (raw and cooked in a steamer) than in conventionally grown potatoes. TE and AA contents showed a significant positive correlation, but only in potatoes from the organic system [R^2^ = 0.686]. By contrast, the positive correlation between TE and TPs was observed regardless of the production system. Therefore, we have identified the effects of farming system, cooking method, and flesh color on the contents of bioactive compounds in potato tubers.

## Introduction

Organically grown food has become increasingly popular in recent years. The reasons that drive consumers to buy organic products vary across countries, but concerns about personal health and the nutritional value of food generally predominate over concerns about environmental issues [1, 2]. Although some consumers believe that organically grown foods are nutritionally better than conventionally grown foods and will pay up to twice as much for organic than for conventional foods [3] studies on this issue are often inconsistent, contradictory, and unable to provide scientific proof for this belief [1, 4].

The many studies collected in the meta-analysis [5, 6] indicated statistically significant and meaningful differences in composition between organic and non-organic crops. Moreover, many researchers have focused on the effects of thermal processing on the concentration of different bioactive compounds of potatoes. Some studies have shown significant decreases in total phenolic contents (TPs) and antioxidant activity in cooked tubers when compared to raw ones [7,8], but other studies reported increases following cooking [9].

The term ‘nutritional value’ refers to the chemical composition of food, in particular to the amounts of key compounds that are essential for functioning of human organism [10]. For example, in potatoes, ascorbic acid (AA), also known as vitamin C, and several secondary metabolites, including phenolic compounds, affect nutritional quality [11, 12]. AA supports the human immune system, plays important roles as an enzyme cofactor in many metabolic functions, and enhances the bioavailability of non-heme iron in humans [13]. Chlorogenic acid is a key phenolic compound that constitutes up to 80% of the TPs in potato [14]. Phenolic compounds benefit human health due to their antibacterial, anti-inflammatory, antiviral, anticarcinogenic, and other properties [15, 16]. AA and phenolics have antioxidant properties and therefore protect cellular constituents against oxidative damage and limit the harmful effects of oxidative stress.

The contents of AA and TPs in potato tubers depend on the variety [17, 18]; potato varieties with purple or red flesh have 3 to 4 times more phenolic acids than do white-fleshed varieties [19] and varieties with purple flesh containabout 1.4 times less AA than traditional yellow-fleshed varieties [20]. Potatoes with coloured flesh also have higher antioxidant activity than do potatoes with white flesh [18], most likely due to the presence of anthocyanins (besides the presence of phenolic acids) [14]. The contents of ascorbic acid and/or TPs of potatoes can be modified by growing conditions, farming system, and nutrient supply [6, 21, 22, 23]. Moreover, the chemical composition and physical characteristics of particular vegetables change during cooking [8, 24]. However, the changes in AA, TPs, and antioxidant activity vary widely according to cooking method [9, 25, 26, 27].

According to Brandt [28], the major problem for researchers (which make difficult comparison of organic and conventional food) is to understand and manage the complexity of the issue, in the context of a general paucity of precise knowledge about the impact of food composition on health. Each study must be designed to provide information in its own right, and to give hypotheses that can be tested in subsequent studies.

In this study, we aimed to investigate the effect of different cultivation systems, cooking methods, and flesh color on AA and TP contents, and antioxidant activity in potato tubers.

## Materials and Methods

### Chemicals

2,6-dichloroindophenol (puriss p.a 97.0%), oxalic acid (puriss p.a ≥99.0%), acetone solution (puriss p.a 99,5%), L-ascorbic acid (L-AA) standard solution (puriss p.a ≥90%), Trolox ((±)-6-hydroxy 2,5,7,8-tetramethylchroman-2-carboxylic acid (97%), 2,2-azinobis(3-ethyl-benzothiazoline-6-sulfonic acid) (ABTS) (activity 90–110%), potassium persulfate (puriss p.a 98%), ethanol (puriss p.a 96%), Folin–Ciocalteu reagent, chlorogenic acid (puriss p.a ≥98.0%), sodium carbonate (puriss p.a 99%). All reagents were from Sigma Aldrich, Fluk, Poch, or Linegal Chemicals.

### Plant material

Potatoes (*S*. *tuberosum* L.) were grown in experimental fields (using organic and conventional systems) at the experimental station of the Plant Breeding and Acclimatization Institute, Research Division Jadwisin, Poland, in 2012 and 2013. Agronomic inputs in organic and conventional systems are shown in Table 1.

**Table 1:** Agronomic inputs in organic and conventional systems.

Crop production practice	Organic system	Conventional system
**Fertilization**	Manure– 28 t.ha^-1^ + mustard as a catch crop	4–5 t plowed rye straw + 1 kg mineral nitrogen per 100 kg straw + mustard as a catch crop;N: 100 kg.ha^-1^, P: 53 kg.ha^-1^, K: 150 kg.ha^-1^
**Weed control**	Only mechanical tillage	Mechanical tillage + herbicides: 2012: Afalon-1.9 l/ha, Titus+Trend (60 g/ha + 0.5 l/ha) 2013: Linurex-1.8 l/ha, Titus + Trend (60 g/ha + 0.5 l/ha)
**Colorado potato beetle control**	Biological insecticide (*Bacillus thuringiensis*) 2012, 2013: 2 times per season-4 l/ha	Chemical insecticides: 2012: Actara -60 g/ha 2013: Actara 2 times per season -70 g/ha, Apacz-40 g/ha
**Late blight control**	Copper fungicides, 2 times per season	Chemical fungicides 2012: Ridomil-2 l/ha, Revus-0.6 l/ha, Ranman-0.2 l/ha, Altima-0.4 l/ha, Ranman-0.2 l/ha, 2013: Revus-0.6 l/ha

Four cultivars (Bursztyn, Ametyst, Flaming, and Stasia) with white, creamy, light yellow, and yellow flesh were evaluated. Seed tubers were obtained from two Polish breeding stations (Zamarte and Strzekęcin) (Table 2). Fifteen-kilogram field samples of tubers from the two farming systems were collected and a laboratory average sample was separated from each field sample and used for further analyses. Only mechanically undamaged tubers of 50 mm in diameter were used for analyses. A laboratory sample (ca. 5 kg, which corresponded to about 100 tubers; size 50 mm) consisted of randomly selected potato tubers. All operations during sample preparation were performed quickly to avoid degradation. Cooking experiments were conducted after harvest and were performed in three technical replications.

**Table 2:** Characteristics of potato cultivars.

Cultivar	Origin of seed tubers	Maturity	Skin color	Flesh color	Average yield [t ha^-1^]^1^	Cooking type^2^
**Bursztyn**	Poland	Mid early	Beige light	White	48.2	BC
**Ametyst**	Poland	Mid early	White yellow	Creamy	64.4	BC
**Flaming**	Poland	Very early	Red	Light yellow	55.6	B
**Stasia**	Poland	Mid early	Yellow	Yellow	33.3	B

^1^ Results from field experiment in 2012–2013

^2^ BC–medium floury, C–floury

### Sample preparation

The following treatments were used prior to analyses of tubers: 1) fresh unpeeled, uncut tubers were boiled in water in a beaker (standard proportions of 0.5 kg of potatoes and 0.7 dm^3^ of boiling water without added salt), for about 15 min ± 2 min (from start point- inserting the tubers into the boiling water); 2) uncut, unpeeled tubers were baked in a microwave oven at 750 W for about 10 min, and 3) uncut, unpeeled tubers were steamed in a steamer at 900 W for about 15 min.

### Measurement of ABTS radical-scavenging activity

ABTS radical-scavenging activity of the hydrophilic fractions was determined by a procedure reported by Rice-Evans et al. [29], using the modifications described by Re et al. [30].

The ABTS_+ solution was prepared by mixing 7 mM of ABTS salt with 2.45 mM of potassium persulfate (final concentration) in 25 ml of distilled water and allowing the mixture to stand in the dark at room temperature for 12–16 h before use. The ABTS_+ solution was diluted with 95% ethanol (approximately 600 μl ABTS to 40 ml 95% ethanol), to obtain an absorbance of about 0.7 (±0.02) at 734 nm. Fresh ABTS_+ solution was prepared for each analysis. Antioxidant or standard solutions (20 μl) were mixed with 1 ml of diluted ABTS_+ solution and incubated at 30°C. The absorbance at 734 nm was read every minute for 30 min. Ethanol (95%) was used as a blank. Trolox, from 0 to 500 μM, was used as a standard. The free radical scavenging activity was expressed as μmoles of Trolox per 100 gram of sample (μmol TE∙100 g^-1^).

### Measurement of total phenolics

Total phenolic content was measured using the modified Folin–Ciocalteu method [31]. The hydrophilic extract (0.5 ml) was diluted with distilled water to 5 ml, to which 0.5 ml Folin–Ciocalteu reagent was added and allowed to react at room temperature for 3 min. After the addition of 1 ml of 1 N sodium carbonate, the mixture was incubated at room temperature for 1.5 h. The absorbance was measured at 725 nm using a spectrophotometer (T70+ UV/VIS) with distilled water as a blank. Chlorogenic acid was used as a standard. Total phenolic contents was reported as milligrams per gram dry matter sample (mg TPs∙g^-1^ DM).

### Measurement of ascorbic acid

AA concentrations were evaluated by a standard spectrophotometric method (Polish standard PN-A04019) [32], based on the ability of AA to reduce the dye 2,6-dichloroindophenol. Briefly, a 10-g laboratory sample of potato tuber was extracted with a solution of 0.4% oxalic acid by homogenizing the sample in an Ultra Turrax T25 for 3 min at 13,500 rpm. The extract was filtered under vacuum through filter paper and brought to 100 ml with the same extraction solution. Next, 5 ml of the extract was reacted with 2 ml of 2,6-dichloroindophenol (1.6%) for 2 min. The absorbance was measured at 500 nm using a spectrophotometer (T70+ UV/VIS) with oxalic acid and 2 ml of 2,6-dichloroindophenol (1.6%) as a blank. The AA concentration was quantified by comparison with a standard curve of L-AA. AA content was reported as milligrams per gram dry matter sample (mg AA∙g^-1^ DM).

### Statistical analysis

Two-way analysis of variance according to constant model and multiple regression analysis with determination of value of the determination coefficients R^2^ was conducted to evaluate if studied factors significantly differentiated the analyzed features. Significant differences between means for the objects (after confirmation of existence of these differences using F-test in analysis of variance) indicated using Tukey`s multiple comparison procedure with P≤0.05. Calculations were done using SAS Statistical Analysis System (v. 8.1) program.

## Results and Discussion

### Ascorbic acid

For potatoes with different flesh colors and grown under two different farming systems, measurements of ascorbic acid (AA) content in raw potatoes and potatoes cooked by three methods (boiled, steamed, or baked in a microwave oven) are given in Table 3. Flesh color had a striking influence on the AA content of potatoes. The AA content for yellow-fleshed potatoes was 1.020 mg∙g^-1^ DM for conventionally grown potatoes and 0.933 mg∙g^-1^ DM for organically grown potatoes.

**Table 3:** Effect of different cooking methods and flesh color on ascorbic acid contents [AA mg∙g^-1^ DM] in potato tubers grown under organic and conventional systems.

Flesh color of potato tubers	Farming system									
	Conventional					Organic				
	Raw potatoes	Boiled	Steamed	Baked in microwayve	*Mean of flesh color*	Raw potatoes	Boiled	Steamed	Baked in microwayve	*Mean of flesh color*
**White**	0.812 ±0.01	0.907 ±0.02	1.058 ±0.05	0.675 ±0.05	0.863^bc^	0.686 ±0.02	0.705 ±0.09	0.887 ±0.01	0.700 ±0.02	0.744^a^
**Creamy**	0.633 ±0.02	0.825 ±0.03	0.874 ±0.07	0.651 ±0.01	0.745^a^	0.633 ±0.02	0.872 ±0.03	0.803 ±0.03	0.751 ±0.02	0.764^a^
**Light yellow**	0.767 ±0.04	0.793 ±0.08	0.926 ±0.06	0.794 ±0.09	0.820^b^	0.792 ±0.03	0.813 ±0.05	0.881 ±0.09	0.665 ±0.06	0.787^a^
**Yellow**	1.020 ±0.04	1.189 ± 0.05	0.939 ±0.03	0.596 ±0.04	0.936^c^	0.933 ±0.01	0.942 ±0.05	0.982 ±0.04	0.880 ±0.01	0.934^b^
*Mean of cooking methods*	0.808^A^	0.928^c^	0.949^c^	0.679^a^		0.761^A^	0.833^ab^	0.888^b^	0.749^a^	
*Mean of growing system*	0.841^a^					0.807^a^				

± standard error of the mean

Mean values denoted by the letters (a, b, c and A,B,C) are statistically significant P≤0,05.

Our studies showed a difference in AA contents between potatoes with white (0.686 mg ∙ g^-1^ DM) and yellow (0.933 m∙ g^-1^ DM) flesh color, grown in an organic system. By contrast, Hejtmánková et al. [20] found that tuber flesh color did not affect significantly the AA contents of potatoes. The contents of vitamin C in raw tubers from both cultivation systems (0.808 mg g^-1^ DM and 0.761 mg g^-1^ DM in conventional and organic systems, respectively) was similar (no statistically significant differences were found Table 4). Smith-Spangler et al. [2] also did not find any significant differences in the content of AA between organic and conventional plants in the review study. Lee and Kader [23] suggested that decrease of AA in many fruits and vegetables may be due to nitrogen fertilization, especially at high rates.

**Table 4:** Sources of variation and ANOVA results „F” for effect of different cooking methods and flesh color on ascorbic acid contents (see Table 3).

Sources of variation	ANOVA results „F”
**Farming system**	4.598 n.s.
**Cooking method**	35.198 *
**Flesh color**	24.098 *
**Farming system x cooking method**	5.181 n.s.
**Farming system x flesh color**	3.722 n.s.
**Farming system x raw potatoes**	1.460 n.s
**Cooking method x flesh color**	5.613 n.s.
**Farming system x cooking method x flesh color**	4.216 n.s.

n.s. not significant

*, significant P≤0.05

In the potatoes grown in a conventional system, we also observed differences in tubers with cream- and yellow-colored flesh (cream, 0.633 mg g^-1^ DM; yellow, 1.020 mg g^-1^ DM). In the organically grown potatoes, the AA contents of potatoes with white- or cream-colored flesh was 26.5% less than the AA contents of potatoes with yellow flesh. In the conventional system, the differed between the AA contents of the yellow- and cream-colored potatoes was about 0.387 mg g^-1^ DM.

The cooked potatoes showed significant variability in AA levels (Table 4). The highest AA contents were measured in potatoes cooked in the steamer (0.949 mg∙g^-1^ DM in the conventional system and 0.888 mg∙g^-1^ DM, organic system) and by boiling in water (0.928 mg∙g^-1^ DM, conventional system and 0.833 mg∙g^-1^ DM organic system) (Table 3). Potatoes cooked in the microwave had significantly lower AA contents (about 0.679 and 0.749 mg∙g^-1^ DM). When comparing the AA content of cooked potatoes (steamed and microwaved) with that of raw potatoes, we found higher losses of the vitamin in conventionally grown potatoes (0.270 mg∙g^-1^ DM less AA in cooked potatoes compared with raw) than in organically grown potatoes (0.140 mg∙g^-1^ DM less AA). Our observations are consistent with the results of Lachman et al. [33], who found higher losses of AA in yellow-fleshed than white-fleshed potato varieties, but only after microwaving. Gołaszewska and Zalewski [34] found that dry methods of cooking potatoes retained AA better, compared with wet methods, because they reduced the AA content by only 8–17%, while wet methods reduced the AA content by up to 40%. This phenomenon likely results from leaching of vitamins during cooking in water, due to the water-solubility of AA [35]. Lower AA levels, observed after microwaving or using other dry methods, can be associated with loss of water content in potatoes. Burg and Fraile [24] found that low water content and the presence of oxygen near the food were responsible for AA losses during prolonged cooking in an oven. In our study, the highest AA content regardless of cooking method (boiled, steamed, or baked in the microwave) was observed for yellow potatoes grown in organic and conventional systems.

Rembiałkowska [10] claimed that organic plant products had higher AA contents than did conventional products. However, we did not find significant differences in AA levels between organically and conventionally grown raw potatoes, regardless of the color of the flesh. Soltoft et al. [12] suggest that differences in the chemical composition of organically and conventionally grown plants could be due to fertilization management.

### Total phenolics

In agreement with our study, numerous studies [36,37] have indicated that plants from organic farms have higher levels of antioxidant compounds than do plants from conventional farms. In the raw tubers of conventionally grown potatoes, TP levels were significantly lower (1.86 mg TPs∙g^-1^ DM) than in tubers of organically grown potatoes (2.033 mg TPs∙g^-1^ DM) (Table 5). Similar results were obtained by Lombardo et al. [38]. However the authors found that TPs show an effect of environment (accounting for 66% of the variance). The environment x cultivar interaction is very important, because it shows that the cultivars responded differently to the environmental conditions. Moschella et al. [39] suggested that the higher accumulation of phenolic compounds in organically grown plants might result from stronger ‘pathogenic pressure’ on organic plants and the resulting increase in the biosynthesis of phenolics. While Brandt and Mølgaard [4] noticed that in an agricultural context (organic), a decrease in nitrogen availability to the plants will result in increased contents of phenolic defense compounds, which then increases the resistance of the plants to pests and diseases.

**Table 5:** Effect of different cooking methods and flesh colors on total phenolic contents [mg TPs g^-1^ DM] in potato tubers grown under organic and conventional systems.

Flesh color of potato tubers	Farming system									
	Conventional					Organic				
	Raw potatoes	Boiled	Steamd	Baked in microwaye	*Mean of flesh color*	Raw potatoes	Boiled	Steamed	Baked in microwaye	*Mean of flesh color*
**White**	0.862 ±0.10	2,085 ±0.08	1.795 ±0.07	2.745 ±0.03	1.871^a^	1.677 ±0.09	1.976 ±0.02	1.984 ±0.02	3.011 ±0.09	2.162^a^
**Creamy**	2.118 ±0.05	2.701 ±0.14	2.360 ±0.10	3.823 ±0.12	2.752^ab^	1.883 ±0.07	2.280 ±0.07	2.334 ±0.04	3.662 ±0.04	2.539^a^
**Light yellow**	1.803 ±0.20	2.261 ±0.02	2.107 ±0.07	2.857 ±0.10	2.257^a^	1.839 ±0.18	2.305 ±0.02	2.134 ±0.02	2.391 ±0.06	2.167^a^
**Yellow**	2.680 ±0.05	2.786 ±0.10	2.936 ±0.10	3.783 ±0.02	3.046^b^	2.734 ±0.07	3.221 ±0.08	3.239 ±0.12	3.512 ±0.10	3.176^b^
*Mean of cooking methods*	1.865^A^	2.458^ab^	2.299^a^	3.302^b^		2.033^B^	2.445^ab^	2.422^ab^	3.144^b^	
*Mean of growing system*	2.481^a^					2.511^a^				

±, standard error of the mean

Mean values in columns denoted by the letters (a, b, c) are statistically significant P≤0,05.

By contrast, Brazinskiene et al. [40] found that the farming system had no significant effect on phenolic acids. Analysis of variance in their studies showed that the phenolic acid contents in potato tubers significantly depends on the variety and the year of the study. This indicates that climatic conditions have a strong effect on biochemical processes in plants and on the contents of biologically active compounds [40]. Lombardo et al. [38] confirmed this and propose that these differences may be due to the use of different varieties of potato or to differences in weather conditions during the growing season.

Our data indicate that potato tuber flesh color had a significant effect on TP contents (Table 6). We observed the highest TPs, regardless of the production system and cooking method used, in tubers with yellow flesh, while tubers with white-colored flesh had significantly lower decreases in TPs with cooking, from a decrease of 0.329 mg TPs∙g^-1^ DM (14%) for steaming to 1.255 mg TPs∙g^-1^ DM (38%) for microwaving or boiling for the organically grown potatoes and from a decrease of 0.689 mg TPs∙g^-1^ DM (27%) for steaming or boiling to 38% for steaming for the conventionally grown potatoes) (Table 5). These relationships were consistent with the results of Lachman et al. [33, 41] and Tierno al. [42].

**Table 6:** Sources of variation and ANOVA results „F” for effect of different cooking methods and flesh colors on total phenolic contents in potato tubers (see Table 5).

Sources of variation	ANOVA results „F”
**Farming system**	0.375 n.s.
**Cooking method**	124.847 *
**Flesh color**	104.791 *
**Farming system x cooking method**	2.363 n.s.
**Farming system x flesh color**	5.542 *
**Farming system x raw potatoes**	5.946 *
**Cooking method x flesh color**	5.624 *
**Farming system x cooking method x flesh color**	2.28 n.s.

n.s. not significant

*, significant P≤0.05

All cooking methods significantly increased or decreased TP contents in potato tubers as compared with raw potatoes (Table 6). On average, the highest TPs was observed in tubers baked in the microwave (organic system, 3.302 mg TPs∙g^-1^ DM; conventional system, 3.144 mg TPs∙g^-1^ DM) and the lowest in tubers cooked by a wet method (boiled or steamed, independent of production system) (Table 5). In the study by Navarre et al. [43] TP levels did not change or decreased after cooking (microwaving, steaming, baking, or boiling). Our work showed similar findings. The largest increase of TP contents in potatoes after cooking was recorded in tubers baked in a microwave, an increase of 1,437 mg TPs g^-1^ DM in potatoes from the conventional system and 1,111 mg of TPs g^-1^ DM in potatoes from the organic system. Compared with the dry method, in the wet methods (boiled and steamed), the increase in TPs was 50% less (about 0.5 mg of TPs g^-1^ DM for boiled and 0.3 mg TPs g^-1^ DM for steamed). This is in agreement with the findings of Blessington et al. [9], who reported that baked, fried, or microwaved potatoes had higher TP contents compared with raw. This study supports numerous other reports [44, 45, 46]. According to the authors, this phenomenon may result from the higher extractability of phenolic compounds from the cellular matrix of potatoes due to changes in starch texture that occur during cooking.

In our study, use of a wet method (boiling in water) lowered the TP contents in all cooked potatoes (organically and conventionally grown), when compared to raw potatoes. This can be explained by the fact that polyphenolic antioxidants are water-soluble and therefore, like AA, can be leached from vegetables by cooking water during boiling [35]. This is consistent with previous studies [7, 25]. However, other studies have shown that heat treatment significantly reduced the TP contents of all vegetables [46].

### Antioxidant activity

The antioxidant activity (in Trolox equivalents, TE) of raw potato tubers grown organically was significantly higher than that of tubers grown conventionally (Table 7). The exception to this was potatoes with white flesh, which showed similar activity (20.20 μmol TE∙100g^-1^). Until recently, conventional wisdom held that processing destroyed natural antioxidants and decreased the antioxidant activity of food. However, many studies indicate that processing does not consistently affect free-radical scavenging activity. A decline of natural antioxidants in the food may be associated with their increased antioxidant activity due to better availability of other antioxidants. Velioglu et al. [47] found high antioxidant activity of the phenolic acids in white-fleshed potatoes, but comparatively low contents of TPs (437 mg per 100 g). Similarly, Kaur and Kapoor [48] observed that tomato, broccoli, Brussels sprouts, turmeric, and lotus had moderate or low TP contents, but high antioxidant activity.

**Table 7:** Effect of different cooking methods and flesh color on antioxidant activity [μmol TE∙100g^-1^] in potato tubers grown under organic and conventional systems.

Flesh color of potato tubers	Farming system									
	Conventional					Organic				
	Raw potatoes	Boiled	Steamed	Baked in microwayve	*Mean of flesh color*	Raw potatoes	Boiled	Steamed	Baked in microwayve	*Mean of flesh color*
**White**	20.20 ±5.14	25.50 ±2.81	33.60 ±0.95	32.70 ±1.91	28.00^a^	20.20 ±8.90	27.20 ±0.75	47.20 ±1.55	33.30 ±1.70	31.97^ab^
**Creamy**	28.30 ±7.04	21.00 ±1.52	36.40 ±2.53	34.90 ±2.48	30.15^a^	40.70 ±9.60	31.70 ±0.41	48.80 ±2.00	34.96 ±2.61	39.04^b^
**Light yellow**	23.30 ±8.17	21.10 ±1.05	31.00 ±1.52	37.20 ±1.17	28.15^a^	37.40 ±9.06	46.35 ±0.80	42.20 ±1.90	30.81 ±4.35	39.19^b^
**Yellow**	27.70 ±5.68	28.30 ±1.15	39.40 ±2.24	35.50 ±2.76	32.72^ab^	40.35 ±5.77	23.45 ±0.55	47.40 ±1.28	40.10 ±4.55	37.82^b^
*Mean of cooking methods*	24.87^A^	23.97^a^	35.10^b^	35.10^b^		34.66^B^	26.57^a^	46.40^c^	34.72^b^	
*Mean of growing system*	29.75^a^					37.00^b^				

±, standard error of the mean

Mean values denoted by the letters (a, b, c and A,B,C) are statistically significant P≤0,05.

Statistical analysis of the results showed a close correlation between antioxidant activity, farming type, and cooking method (Table 8). We measured the highest antioxidant activity in organically grown potatoes cooked in a steamer (46.40 μmol TE∙100 g^-1^) (Table 7). Organic and conventional potatoes cooked in the microwave oven showed comparable TE values (about 35 μmol TE∙100 g^-1^) and potatoes cooked by boiling showed significantly decreased antioxidant activity, regardless of the farming system used. Although it is difficult to compare results of different studies due to usage of different antioxidant activity assays, in contrast to our results, Brown et al. [49] found that boiling contributed to a much greater increase of antioxidant activity determined by the H-ORAC method, as compared with frying, baking, or microwaving of potatoes. Also, Perla et al. [7] found that boiling, microwaving, and baking reduced free radical scavenging activity by 26%, 32%, and 38%, respectively. Nebesny and Budryn [26] found that heating in the microwave oven results in smaller losses of antioxidant activity of food than does convection heating. Blessington et al. [9] observed a greater increase in antioxidant activity (quantified as μg Trolox equivalents per g fresh weight) after microwaving and frying than after baking and boiling.

**Table 8:** Sources of variation and ANOVA results „F” for effect of different cooking methods and flesh color on antioxidant activity in potato tubers (see “Table 7”).

Sources of variation	ANOVA results „F”
**Farming system**	63.214 *
**Cooking method**	39.239 *
**Flesh color**	6.677 *
**Farming system x cooking method**	8.063 *
**Farming system x flesh color**	3.24 *
**Farming system x raw potatoes**	4.613 *
**Cooking method x flesh color**	4.979 *
**Farming system x cooking method x flesh color**	4.915 *

*, significant P≤0.05

### Relationship between ascorbic acid, total phenolic contents and antioxidant activity

**Fig 1:**
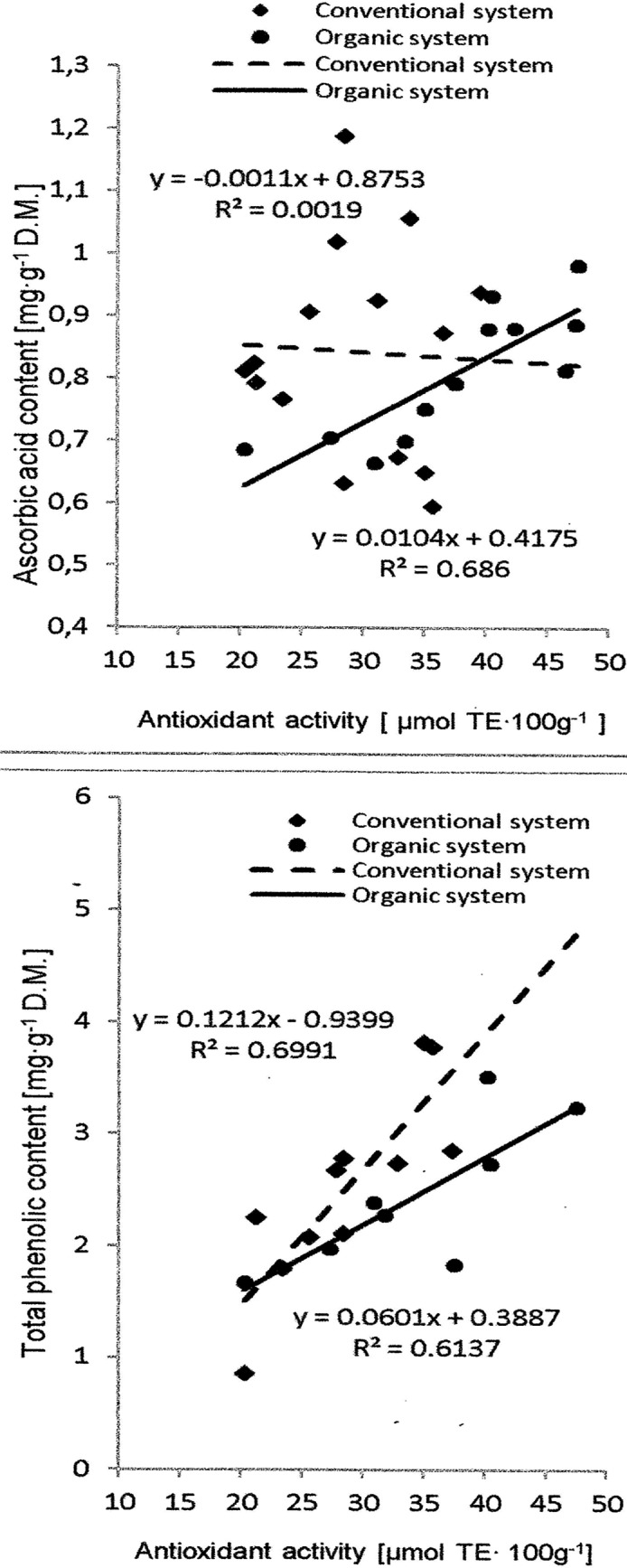
The relationship between antioxidant activity and bioactive compounds in potato tubers grown in two production systems.

The relationship between antioxidant activity in potato tubers and AA or TPs from two production systems is shown in “Fig 1”.

We observed that for the potatoes grown in the organic system, the higher the AA contents, the higher the antioxidant activity “Fig 1”. The correlation coefficient between the antioxidant activity and AA was R^2^ = 0.686. We found no correlation for potatoes grown in the conventional system (R^2^ = 0.001). Hejtmánková et al. [20] also found no significant correlation between AA contents and antioxidant activity (R^2^ = 0.08) in potatoes grown in a conventional system. For phenolics, the correlation between TP contents and antioxidant activity is well known [50, 51, 52, 53]. The correlation coefficients between TPs and TE measured in the aforementioned studies ranged from 0.430 [53] to 0.879 [51]. In our study, the correlation coefficients between antioxidant activity and TP contents were R^2^ = 0.6991 for potatoes grown in the conventional system and R^2^ = 0.6137 for potatoes from the organic system (insignificant differences) “Fig 1”. Similarly, Nzaramba et al. [50] found that total phenolic contents is positively correlated with antioxidant activity (correlation coefficients 0.579 for DPPH and 0.876 for ABTS).

## Conclusions

We conclude that the AA contents of potato tubers depends on the flesh color and cooking method used. The yellow-fleshed potatoes grown conventionally and cooked in the microwave showed the greatest losses of AA (0.424 mg of TPs g^-1^ DM). In potatoes from the organic system, we did not observe such losses of AA. The dry method of cooking (baking in the microwave) increased the TP contents in potatoes by about 30% regardless of the flesh color and the production system. Antioxidant activity was significantly higher in organically grown potatoes (raw and cooked in a steamer), than in potatoes from the conventional system. Also, antioxidant activity and AA contents showed a significant correlation, but only in potatoes from the organic system [R^2^ = 0.686]. We observed a positive correlation between antioxidant activity and TPs regardless of the production system. Therefore, our observations indicate substantial effects of both flesh color and cooking method; however, the effects of cultivation system differed for different conditions.

## Abbreviations

AA: ascorbic acid
DM: dry matter
TPs: total phenolics
TE: antioxidant activity, Trolox equivalents
